# *Scenedesmus rubescens* Heterotrophic Production Strategies for Added Value Biomass

**DOI:** 10.3390/md21070411

**Published:** 2023-07-19

**Authors:** Gonçalo Espírito Santo, Ana Barros, Margarida Costa, Hugo Pereira, Mafalda Trovão, Helena Cardoso, Bernardo Carvalho, Maria Soares, Nádia Correia, Joana T. Silva, Marília Mateus, Joana L. Silva

**Affiliations:** 1 Allmicroalgae Natural Products S.A., R&D Department, Rua 25 de Abril s/n, 2445-413 Pataias, Portugal; moisesgoncalo.16@gmail.com (G.E.S.); ana.barros@allmicroalgae.com (A.B.); mafs.8@hotmail.com (M.T.); helena.cardoso@allmicroalgae.com (H.C.); marjsoares@gmail.com (M.S.); nadiasgcorreia@gmail.com (N.C.); joanatlfsilva@gmail.com (J.T.S.); joana.g.silva@allmicroalgae.com (J.L.S.); 2 Microalgae Section, Norwegian Institute for Water Research (NIVA), Økernveien 94, 0579 Oslo, Norway; costa.anamarg@gmail.com; 3 GreenCoLab—Associação Oceano Verde, University of Algarve, Campus de Gambelas, 8005-139 Faro, Portugal; hugopereira@greencolab.com (H.P.); bernardocarvalho@greencolab.com (B.C.); 4 iBB—Institute for Bioengineering and Biosciences, Instituto Superior Técnico, Universidade de Lisboa, Av. Rovisco Pais, 1049-001 Lisbon, Portugal

**Keywords:** *Scenedesmus rubescens*, heterotrophy, media optimization, stirred-tank reactor, DoE—design of experiment, RSM—response surface methodology

## Abstract

Microalgae attract interest worldwide due to their potential for several applications. *Scenedesmus* is one of the first in vitro cultured algae due to their rapid growth and handling easiness. Within this genus, cells exhibit a highly resistant wall and propagate both auto- and heterotrophically. The main goal of the present work is to find scalable ways to produce a highly concentrated biomass of *Scenedesmus rubescens* in heterotrophic conditions. *Scenedesmus rubescens* growth was improved at the lab-scale by 3.2-fold (from 4.1 to 13 g/L of dry weight) through medium optimization by response surface methodology. Afterwards, scale-up was evaluated in 7 L stirred-tank reactor under fed-batch operation. Then, the optimized medium resulted in an overall productivity of 8.63 g/L/day and a maximum biomass concentration of 69.5 g/L. *S. rubescens* protein content achieved approximately 31% of dry weight, similar to the protein content of *Chlorella vulgaris* in heterotrophy.

## 1. Introduction

Microalgae or microphytes are microscopic ancestral living organisms defined as oxygenic photosynthesizers. These organisms comprise over 300,000 species of which approximately 30,000 are documented [1]. Their potential to be used in wastewater treatment and effluent bioremediation has been widely discussed [1], as well as other uses, namely for food and feed applications and added value compound extraction [2].

To overcome prohibitive production costs and to achieve the high purity required for more refined niche markets (such as cosmetics and pharmaceutical industries), it is possible to use biorefinery approaches to extract a wide variety of bioproducts, such as proteins, carbohydrates, carotenoids, and lipids such as DHA (docosahexaenoic acid) and EPA (eicosapentaenoic acid) [1]. Besides being more readily incorporated into commonly used products than whole biomass, microalgae extracts are functional ingredients, conveying bioactive properties to those products [3]. Therefore, it is possible to take full advantage of microalgae’s inherent ability to produce valuable compounds, channel the different fractions into specific applications directed at highly refined markets, and make the whole production process economically viable [4].

Microalgae can be produced under autotrophic, mixotrophic, and heterotrophic conditions. However, only a few microalgae, such as *Scenedesmus* sp., *Chlorococcum* sp., *Chlorella* sp., and *Chlamydomonas* sp., grow heterotrophically [5,6,7,8]. Under these conditions, microalgae use organic substrates both as energy and as carbon sources [9], and production occurs in closed-stirred reactors, such as industrial fermenters [9], and in axenic conditions [10]. Heterotrophic growth is light-independent and allows faster growth and higher yields. For instance, *Scenedesmus acuminatus* produced heterotrophically yielded 274 g/L of dry biomass [5]. Thus, it decreases the need to occupy large areas for inoculum production [7,9]. Overall, it allows efficient, controlled, reproducible, and reliable year-round production, overcoming major limiting factors of autotrophic cultivation, namely the dependency on weather conditions [11].

The green microalgae *Scenedesmus* sp. (Chlorococcales; Scenedesmaceae) are commonly found in fresh and wastewater streams [12]. These algae are typically characterized by a two-dimensional arrangement of two or more cells in regular aggregates called coenobia [13], and algae from this genus were some of the first cultured in vitro due to their rapid growth and handling easiness [12]. *Scenedesmus* sp., similarly to other coccoid green algae, present highly resistant cell walls exhibiting a characteristic trilaminar structure [14].

*Scenedesmus* sp. can grow both auto- and heterotrophically and has untapped biotechnological potential. They are considered a valuable source of protein, containing up to 60% [15], and, when stress-induced, *Scenedesmus* sp. direct their metabolism to accumulate lipids by repurposing other energetic components, such as proteins and polysaccharides, a key feature for biofuel development [5,16,17]. Lastly, these microalgae can produce carotenoids such as lutein and astaxanthin [18]. This group of pigments is targeted by food, feed, and cosmetic industries due to their appealing color, aroma, remarkable nutritional composition [19,20], and bioactivity as powerful antioxidants [20,21].

To cultivate microalgae and produce a given metabolite, a combination of parameters must be considered [22], namely nutritional or chemical factors and environmental or physical factors. The first includes chemical elements in the culture medium essential for the cell’s metabolism, such as carbon, nitrogen, phosphorus, calcium, sodium, silica, metals such as iron and copper, etc. [23,24]. On the other hand, environmental factors include pH, temperature, agitation, and aeration intensity in the system [23].

Traditionally, culture medium optimization is achieved through an OVAT approach, i.e., “one variable at a time” [25]. Although simple, OVAT becomes time-consuming and inefficient since it does not consider possible interactions between different factors [26]. In addition, this time- and labor-intensive approach comes at increased costs [27] compared to alternative methods such as the design of experiments (DoE). DoE is a statistical performance analysis method that allows the development of a model which can predict some system responses given the change of the variables under study [25]. In addition, DoE determines the importance of the factors (screening) and their interactions (optimization) [28]. It determines the effect of each factor (variable in study) individually or by changing the level of other factors (interactions), which means the level of one factor varies the effect that other factors will have on a specific response [29].

In a complex microbial process, evaluating the interactions between the studied variables is critical for obtaining the optimal operation point. The system responses could be biomass production or biocompound(s) productivity [28].

The present work aimed at obtaining an optimal culture medium to cultivate *Scenedesmus rubescens* under heterotrophic conditions. Medium validation with high cell density and biomass characterization for further potential commercial application were also accessed.

## 2. Results

### 2.1. Growth Performance

#### 2.1.1. Preliminary Assays (Carbon and Nitrogen Sources and Working pH)

Preliminary assays, aiming to find a baseline medium for the optimization study, were performed. First, different carbon sources (glucose, acetate, and glycerol) were tested. Glucose was the only one that promoted cell growth, and since it was already used to grow *Scenedesmus acuminatus* [5], *Scenedesmus obliquus* [30], and *Chlorella vulgaris* [11], this was the chosen carbon source from this point on.

**Figure 1:**
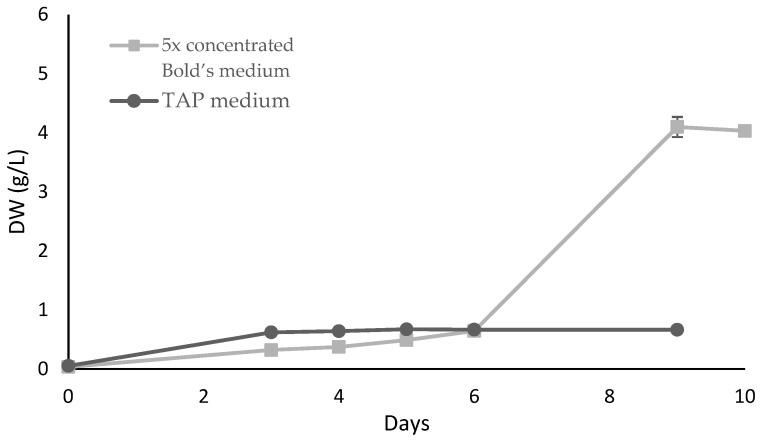
*Scenedesmus rubescens* growth curves under heterotrophic cultivation in 250 mL Erlenmeyer flasks using TAP or 5× concentrated Bold’s media supplemented with 20 g/L glucose. The values represent the average and respective standard deviation of 3 individual experiments. SD values were lower than 0.04 g/L.

Two different media, TAP [31] and 5× concentrated Bold’s medium [32], were screened using OVAT methodology (Figure 1). Both were supplemented with 20 g/L of glucose. The nitrogen sources were ammonia and nitrates for TAP and Bold’s media, respectively.

The highest biomass concentration (4.1 g/L) was reached using Bold’s medium while TAP medium only reached 0.81 g/L, as depicted in Figure 1. Comparing the composition of both media, 5× concentrated Bold’s medium had a higher concentration of most nutrients, particularly nitrogen and phosphate, which could influence *Scenedesmus* growth as it also affected the growth of *Chlorococcum* sp. and *S. acuminatus* in other published studies [5,6].

**Figure 2:**
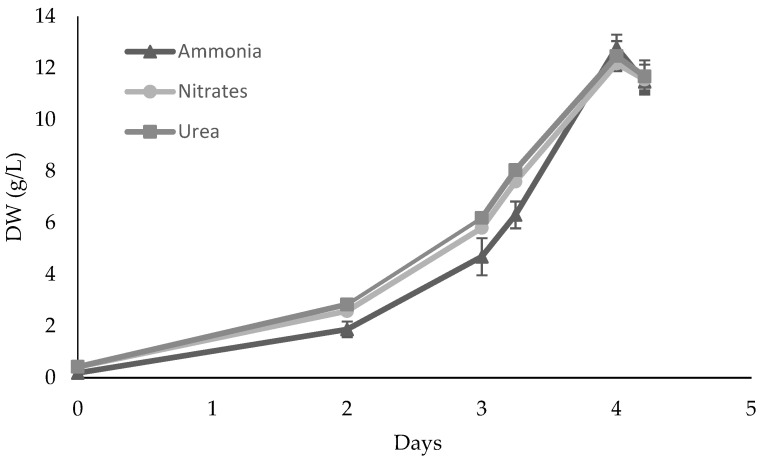
*Scenedesmus rubescens* growth curves using 0037SA medium supplemented with different nitrogen sources and 20 g/L glucose. Cultures were grown heterotrophically in 250 mL Erlenmeyer flasks. The values represent the average and respective standard deviation (SD) of 3 individual experiments. SD values were lower than 0.26 g/L.

Since these media have different nitrogen sources, which could also compromise cell growth [33], the next step was to evaluate *S. rubescens* growth using nitrates (120 mM), ammonia (60 mM), and urea (60 mM) (Figure 2).

No significant differences were found (*p* > 0.05) among treatments, and 13 g/L of dry biomass and 0.91 g/L/day of global productivity were obtained. This result suggests the possibility of using urea and nitrate, which is in agreement with previous studies where *Scenedesmus acuminatus* was supplemented with these two nitrogen sources [5]. However, in this work, ammonia could also be used to control pH in later stages of the scale-up process, suggesting it could also become a promising nitrogen source.

**Figure 3:**
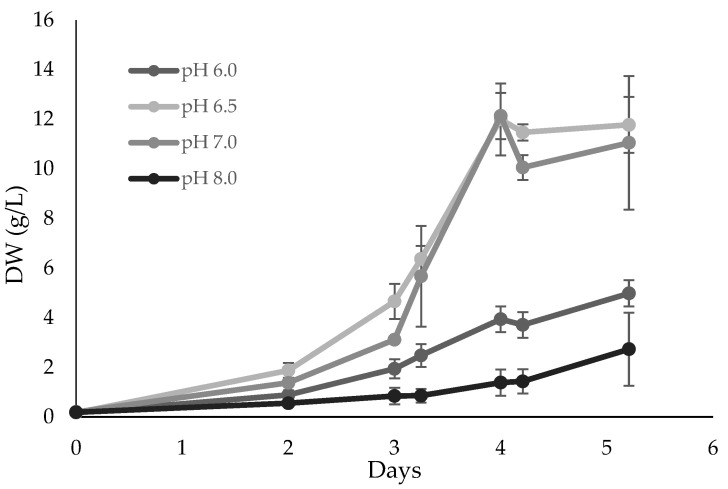
*Scenedesmus rubescens* growth curves using 0037SA medium supplemented with 20 g/L glucose at different pH values. Cultures were grown heterotrophically in 250 mL Erlenmeyer flasks. The values represent the average and respective standard (SD) deviation of 3 individual experiments. SD values are lower than 0.91 g/L.

The pH determines the solubility of nutrients and drives many cellular responses, which can significantly influence overall microalgal metabolism [34]. The optimal pH was, therefore, searched. Four pH values were used during the experiments (6.0, 6.5, 7.0, and 8.0, Figure 3). The pH was maintained using 80 mM of PIPES buffer.

Under pH 6.5 and 7.0, the culture reached higher cell productivity and growth rate (Table 1). Other studies [5] showed *Scenedesmus acuminatus* achieving a higher concentration at pH 6.0. However, *S. rubescens* growth continues to be favored under a weak acidic/neutral pH environment, unlike *S. acuminatus*. Other resemblant heterotrophic species, such as *Chlorella vulgaris*, have also been cultivated at pH 6.5 in 7 L bench-top fermenters [7].

#### 2.1.2. Culture Medium Screening Using Plackett–Burman Design

**Figure 4:**
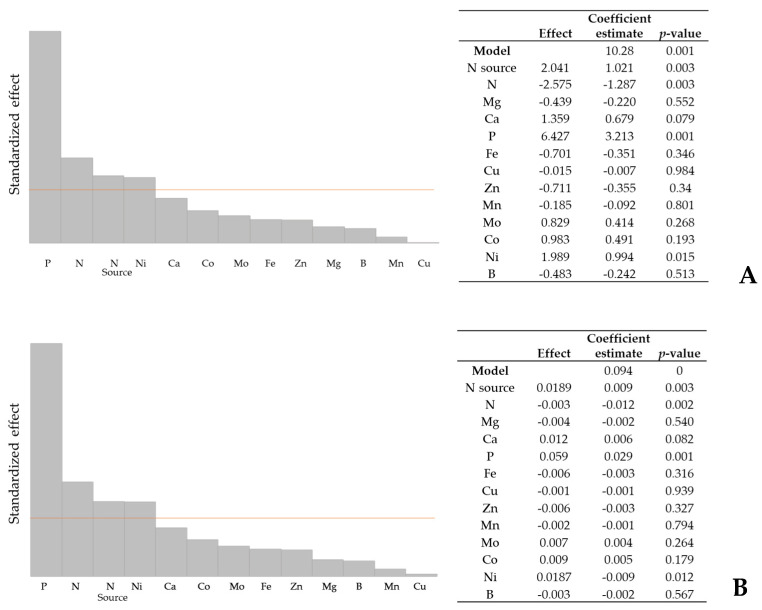

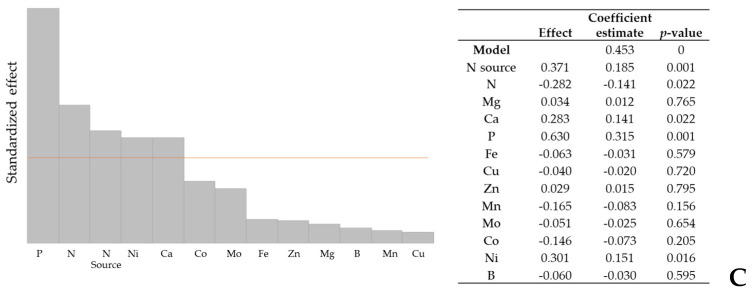
Order bar charts (Pareto charts) and analysis of variance (ANOVA) obtained with the software Minitab^®^ version 19, testing 13 factors for 3 responses: (**A**) biomass concentration, (**B**) global productivity, and (**C**) maximum productivity. Factors above the red line are the most significant factors for all three responses. The model was significant (*p* < 0.05). The cultures were grown heterotrophically in 250 mL Erlenmeyer flasks.

As previously mentioned, nutrients are essential for the growth and development of microalgae. In this way, 12 nutrients were studied under different concentrations: N, Mg, Ca, P, Fe, Cu, Zn, Mn, Mo, Co, Ni, and B. Factors and their concentrations were chosen based on previously tested media (TAP and Bold’s). Screening was carried out to predict which nutrients influence biomass productivity (Figure 4). Nitrogen sources (nitrates and ammonia) were included to understand their influence on/under different concentrations of other nutrients.

The Plackett–Burman design was used with two coded levels, and 30 runs were employed (Table S1) with the chosen responses: (1) biomass concentration, (2) global productivity, and (3) maximum productivity. Low- and high-level concentrations were defined based on the previously studied culture media (Section 2.1.1).

The nitrogen source was one of the most significant factors affecting cell growth (*p* < 0.05). However, in the previous experiment (Figure 2), there was no significant difference between ammonia and nitrates. Therefore, ammonia was chosen given the convenience regarding pH control in later stages of scale-up. The concentrations of N, P, Ni, and Ca also significantly influenced cell growth (Figure 4). However, calcium concentration only affected maximum productivity (Figure 4C).

#### 2.1.3. Culture Medium Optimization Using Box–Behnken Design

**Figure 5:**
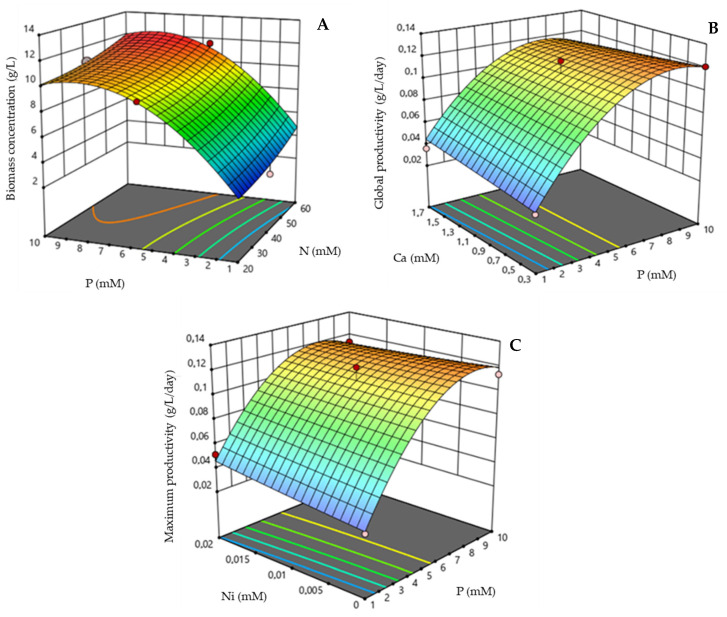
Response surfaces showing the mutual effects of factors present in the culture medium. (**A**) Effects of the interaction between P and N factors for biomass concentration response; Ni was kept at maximum level and Ca was kept at lowest level. (**B**) Effects of the interaction between P and Ca factors for global productivity response; N and Ni were kept at the intermediate levels. (**C**) Effects of the interaction between P and Ni factors for maximum productivity response; N and Ca were kept at the intermediate levels. The cultures were grown heterotrophically in 250 mL Erlenmeyer flasks.

Design-Expert software was used to further optimize the medium composition through Box–Behnken design via the response surface method (RSM). The N, P, Ni, and Ca element concentrations were further optimized (Table 2). In this experimental design, 26 experimental sets were generated with three central points (Table S2). The same responses as before were addressed, including biomass concentration, global productivity, and maximum productivity (Figure 5).

Figure 5 represents the prediction of the interaction among different factors in *S. rubescens* culture medium. In general, the model predicts that P will achieve maximum values to increase all these responses (10 mM). Regarding biomass concentration (Figure 5A), the model shows that P and N concentrations should be near the highest concentrations used (10 and 60 mM, respectively) to achieve higher biomass concentration. The predicted model is represented by Equation (1), *p* < 0.05. Figure 5B characterizes the interaction between Ni and P for global productivity response. N and Ni concentrations at the central values demand P at the highest (10 mM) and Ca at the lowest value (0.3 mM) to obtain the highest global productivity. As a result, the predicted equation was Equation (2) *p* < 0.05.

Finally, P and Ni at the highest level (10 and 0.02 mM, respectively) and N and Ca at the central point induced higher maximum productivity values (Figure 5C). The model predictions are described by Equation (3) (*p* < 0.05).

From the models designed, it was possible to conclude that for an optimized *S. rubescens* culture medium, the highest level for factors N (60 mM), P (10 mM), and Ni (0.02 mM), and lowest value of Ca (0.3 mM) were necessary.

Biomass concentration (g/L):

(1) 9.74−0.0185A+3.58B+0.2010C+0.1098D−0.0268A×B−0.9468A×C+0.2881A×D−0.0473B×C−0.4322B×D−0.5969C×D+0.3750A×A−2.85B×B+0.3790C×C+0.0179D×D

Global productivity (g/L/day):

(2) 0.1101−0.0014A+0.0382B+0.0004C+0.0016D−0.0043A×B−0.0035A×C+0.0091A×D−0.0062B×C−0.0053B×D−0.0040C×D+0.0001A×A−0.0335B×B−0.003C×C+0.0005D×D

Maximum productivity (g/L/day):

(3) 0.269+0.0266A+0.131B+0.001C+0.001D

**Figure 6:**
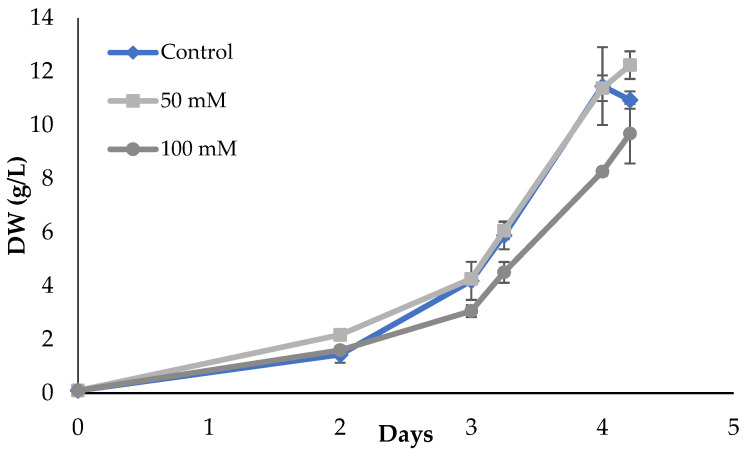
*Scenedesmus rubescens* growth curves using 0037SA medium supplemented with different phosphate concentrations and 20 g/L glucose. Cultures were grown under heterotrophic conditions in 250 mL Erlenmeyer flasks. The values represent the average and respective standard (SD) deviation of 3 individual experiments. SD values are lower than 0.94 g/L.

Finally, to assess the possibility of phosphate being a growth limiting factor, different concentrations were tested, including 10 (control), 50, and 100 mM (Figure 6).

Alga growth led to similar biomass concentration, comparing the use of 10 and 50 mM of phosphate (11.5 to 12.2 g/L; Table 3). Data suggest there are only growth differences with 100 mM of phosphates, possibly caused by the initial inhibition of cell growth (*p* < 0.05). Comparing global productivity and specific growth rate (Table 3), there were no significant differences between the use of 50 mM and 10 mM phosphate, neither between 50 and 100 mM (*p* > 0.05), but there was a significant difference between 10 and 100 mM. Overall, 50 mM of phosphate was used in the following assays.

In this way, when comparing Bold’s medium and 0037SA medium, *S. rubescens* growth was improved by 3.2-fold (from 4.1 to 13 g/L of dry weight), indicating that the medium optimization succeeded.

### 2.2. Validation of Optimized Medium in Bench-Top Fermenters

**Figure 7:**
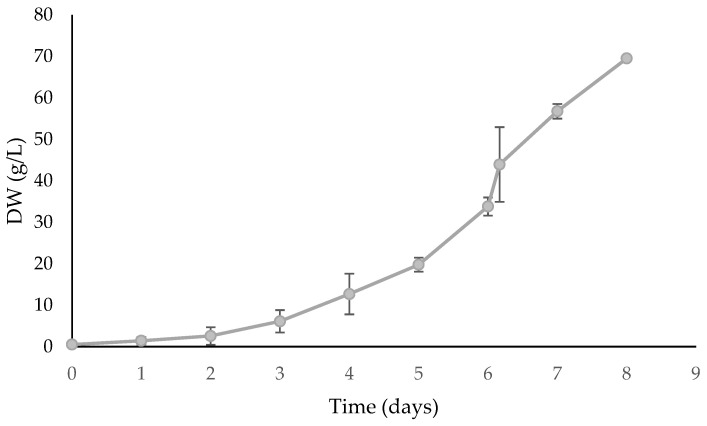
*Scenedesmus rubescens* growth curve in a 7 L bench-top fermenter using the optimized heterotrophic medium. Dissolved oxygen (DO) inside the fermenter was controlled automatically above 40% saturation by increasing aeration up to 5 L/min and stirring speed up to 1200 rpm. The values represent the average and respective standard deviation (SD) of 3 individual experiments. SD values are lower than 2.82 g/L.

The optimized medium resulted in an overall productivity of 8.63 g/L/day and a maximum biomass concentration of 69.5 g/L (Figure 7). This concentration is much higher than what was reported for the same species grown autotrophically, which was 4.1 g/L [33]. However, it is significantly lower than that found for *S. acuminatus* (274 g/L) [5], but the fact that this is a different species should be taken into account. The medium pH in the fermenter from the referenced study was set to 6, rather than 6.5. Additionally, to optimize the biomass concentration, the fermenter feeding was determined by controlling glucose concentration in the range of 0–5 g/L. In the present study, glucose concentration was controlled in the range of 0–20 g/L, and a tighter control may be crucial. Compared to other published data, the cell densities obtained herein represent higher biomass titers than those obtained in other studies with *Aurantiochytrium* sp. (batch) [35], *Chlorella vulgaris* (fed-batch) [7], *Chloroccoccum amblystomatis* (batch) [6], *Nitchia laevis* (fed-batch) [36], and *Schyzochytrium* sp. (fed-batch) [37]. Overall, although different species may behave and respond differently, *S. rubescens* was able to reach a high biomass concentration, in line with other fed-batch heterotrophic microalgae species. Still, further studies are required to optimize *S. rubescens* growth and obtain even higher cell densities and to develop its biotechnology potential for commercial applications.

### 2.3. Biochemical Analysis

The biochemical composition of the biomass obtained during the validation at the beginning and end of the growth curve (initial and final phase) were analyzed, and the content of proteins, lipids, carbohydrates, and ashes was assessed to understand if the different stages influenced biochemical composition (Table 4).

*S. rubescens* biomass displayed 33% and 31% of protein at the initial and final phase of cultivation in the fed-batch fermenter, respectively. These values are comparable to those attained with heterotrophically cultured *Chlorella vulgaris* [7], suggesting that *Scenedesmus* sp. also has great potential to produce biomass for alternative protein markets. Under autotrophic conditions, *S. obliquus*, as other species such as *Chlorella vulgaris* and *Arthrospira platensis*, achieved between 50 and 60% [15], which is significantly higher than *S. rubescens* in heterotrophy. However, heterotrophically produced *S. rubescens* could also subsequently inoculate photobioreactors, where cells would grow autotrophically. This strategy, already used for *Chlorella*, increases production efficiency to obtain a highly concentrated biomass for the inoculation of reactors operating under autotrophic conditions [38] and can be coupled to a second stage of autotrophic cultivation, which would most likely increase the protein and pigment contents and result in higher quality microalgal biomass, as shown in *Chlorella*.

Concerning lipid content, cells grown in the fermenter obtained 13% at the initial and 12% at the final growth phases. This result is in agreement with the lipid content reported in the literature for *S. obliquus* [15]. However, Cheng. et al. 2018 reached 31% [39] by actively inducing lipid production through nitrogen depletion strategies. In another study, also through a nitrogen depletion strategy, *Scenedesmus abudans* achieved high lipid content, between 36% and 67% [40]. Overall, according to the literature, microalgae tend to accumulate lipids when metabolically stressed as a tradeoff of other energetic components, such as proteins and polysaccharides, as reported, for instance, for *Nannocloropsis* sp. [17,41,42].

Lastly, *S. rubescens* obtained a remarkably low ash content (2.3% and 3.2% at initial and final growth phases, respectively) in comparison to that of other microalgae, namely *Arthrospira platensis* (14.5%) [43] and *Nannocloropsis* sp. [44]

Altogether, *S. rubescens* was shown to be a promising source of relevant compounds, such as proteins and lipids, comparable to other commercially available species. Furthermore, depending on the desired commercial application, heterotrophic growth is a promising strategy to obtain high biomass yields or a given metabolite of interest.

## 3. Discussion

To the best of the authors’ knowledge, this represents the first report on *S. rubescens* under heterotrophic conditions. The optimization of culture medium was performed (Table S3) and compared to the initial Bold’s growth medium. When comparing these media, *S. rubescens* growth was successfully improved by 3.2-fold (from 4.1 to 13 g/L of dry weight).

The medium composition resulting from the optimization was also compared to that reported by Jin et al. (2020), designed for *Scenedesmus acuminatus* [5]. While Jin et al. (2020) found the optimum pH at 6.0, the strain used in the present study grew optimally at pH 6.5. In addition, when comparing both media, 0037SA medium is formulated with higher nutrient concentrations, which could have compromised cell growth. *S. acuminatus* is described as reaching a maximum of 274 g/L on a 7.5 L fermenter [5], a biomass concentration value that is significantly higher than the one obtained in the present study. The authors also started to use nitrates (30 mM) as nitrogen sources, and the study was also performed at the laboratory scale; nevertheless, the N-source was replaced by urea at 0.85 g/L in the batch fermenter medium, and the study was concerned with a different species. All these differences most likely influence cell growth significantly.

In a study performed with *Chlorella vulgaris*, biomass reached 175 g/L in a 7 L heterotrophic scale-up phase [7]. Nevertheless, this higher biomass titer was achieved after further medium and abiotic parameter optimization steps.

The optimization strategies in the studies referred to above ([5,7,9]) allow us to hypothesize that there are still opportunities for the further improvement of *Scenedesmus rubescens* biomass productivity in batch and fed-batch bioreactor cultivation such as culture media or growth strategies (C-source concentration control, aeration, or stirring speed, etc.).

Heterotrophically produced *S. rubescens* presented an appealing nutritional profile, and both literature and empirical large-scale production experience suggest there is potential to increase the protein content of this microalgal species under autotrophic conditions. Therefore, one way to develop its commercial application potential could be to combine hetero- and autotrophic cultivation modes, taking advantage of the two metabolic pathways [7,38].

Overall, cultivation conditions were key factors influencing both the growth process and the biochemical profile of the final biomass. Only by learning how to manipulate these variables and understanding the systems’ responses does it become possible to grow uncommon microalgae species. The present work demonstrates pilot-scale feasibility of *S. rubescens* production under heterotrophic conditions, shows the derived microalga proximate composition, and highlights strategies for potential commercial applications. Whether aiming at vegetarian/vegan protein substitutes or lipids for biofuel production [39], studies addressing industrial production feasibility open new routes toward commercial application and bring us one step closer to market viability.

## 4. Materials and Methods

### 4.1. Microalgae Strain and Culture Media

The axenic *Scenedesmus rubescens* used in this work were obtained from Allmicroalgae’s own culture collection (strain code AGF0037SA). This alga was stored in agar slant tubes and subsequently scaled to 250 mL Erlenmeyer flasks. Initially, culture medium was PCB (plate count broth). Throughout experiments, cultures were grown in optimized media. Through the optimization work, the growth medium utilized was continuously updated.

The following media were used for preliminary tests: TAP (Tris-acetate-phosphate) medium [31] and 5× concentrated Bold’s Basal Medium [32]. All media were supplemented with 20 g/L glucose. Lastly, 0037SA medium (Table S3) was created and optimized according to the assays described.

Two types of tests were performed to optimize the culture media: OVAT and DoE tests. All the assays were performed using triplicates, except for DoE tests. All culture media were sterilized using filtration through a 0.2 μm pore size PES membrane in a Vacuum Filtration System (VWR, Radnor, PA, USA) and/or autoclaved (Uniclave88 and uniclave77, A.J.Costa, Irmãos, Lda; Cacém, Portugal) at 121 °C for 40 min.

### 4.2. Growth Assessment

*S. rubescens* growth was determined using optical density (OD) and dry weight (DW). OD was measured at 600 nm (Figure S1) using a spectrophotometer (Genesis 10S UV–Vis -Thermo Scientific, Waltham, MA, USA). DW was determined using the filtration of culture samples with pre-weighed 0.7 μm GF/C 698 filters (VWR, PA, USA) and dried at 120 °C on a DBS 60–30 electronic moisture analyzer (KERN & SOHN GmbH, Balingen, Germany). These measurements were used to study cell growth, namely specific growth rate, and maximum and overall productivities were calculated.

The specific growth rate (*μ*) was calculated according to Equation (4):

(4) $$\mu \;({\mathrm{day}}^{-1})=\frac{\ln\left(X2/X1\right)}{t_{2}-t_{1}}$$

X refers to dry biomass concentration (g/L) at time t_2_ and t_1_ (days) of cultivation within the exponential growth phase.

Volumetric biomass productivity (Pv) was calculated according to Equation (5):

(5) $$\mathrm{Pv}=\frac{\mathrm{Xf}-\mathrm{Xi}}{\mathrm{tf}-\mathrm{ti}}$$

where Xf corresponds to final dry biomass concentration, Xi corresponds to initial dry biomass concentration (g L^−1^), tf corresponds to final time, and ti corresponds to the initial time (h) of cultivation within the exponential growth phase.

### 4.3. Experimental Trials

All experimental trials for medium optimization were conducted in 250 mL baffled Erlenmeyer flasks with vented caps with a 0.2 μm PTFE membrane (Duran™, Munich, Germany) with a working volume of 50 mL. Cultures were grown in an orbital shaker incubator (SKI 4, ARGOLAB, Carpi, Italy) at 28 °C and 200 rpm (revolutions per minute). All assays ended when cultures reached the stationary phase or carbon source depletion.

Initially, two culture media (5× concentrated Bold’s Basal Medium and TAP medium) were tested and the alga’s growths compared. Subsequent cultures with the supplementation of different nitrogen sources (ammonia, nitrates, and urea) at different pH values (6.0, 6.5, 7.0, and 8.0) followed and were analyzed. Based on the outcomes, a screening test was carried out to find the impact of different medium composition factors on S. rubescens propagation. Lastly, a Box–Behnken design was conducted to optimize the final culture medium.

Erlenmeyer flask cultures were further scaled-up to inoculate a 7 L bench-top fermenter (New Brunswick BioFlo^®^/CelliGen^®^115; Eppendorf AG, Hamburg, Germany) to validate the culture medium. Cultures were grown in a fed-batch regime at 28 °C, and pH was maintained at 6.5 by adding ammonia solution (24% *w/w*), also ensuring a nitrogen source. Glucose and phosphate concentrations were measured twice a day throughout the assays and supplemented to guarantee optimal growth conditions. In the case of glucose, a pure sterile solution of 500 g/L was added in pulses to maintain medium concentration within the range of 0–20 g/L. Additionally, a sterile 2.5 M phosphate buffer solution was added to maintain the medium concentration of 50 mM. Dissolved oxygen (DO) inside the fermenter was controlled automatically above 40% saturation by increasing aeration up to 5 L/min and stirring speed up to 1200 rpm. Samples at the beginning (immediately after inoculation) and end of the fermenter operation time (reported) were collected to analyze the biochemical composition of the respective algal biomasses.

### 4.4. Nutrient Quantification

The cultures sampled (50 mL) were centrifuged for 10 min at 3500 rpm in VWR Mini Star microcentrifuge (VWR, Radnor, PA, USA). The supernatant was collected to quantify glucose, phosphate, and ammonium concentrations.

When necessary, the supernatant was diluted in a saline solution (10% sodium chloride, 90% distilled water). Freestyle precision Neo kit (Abbott, Witney, Oxon, UK) was used to determine glucose concentration in g/L.

Ammonia and phosphate Sera Tests (Sera, Heinsberg, Germany) were used to determine ammonium and phosphate concentrations, respectively. The supernatant was diluted with distilled water when necessary. The absorbance was measured at the wavelength of 697 nm for ammonium and 716 nm for phosphate. The absorbances were measured using Genesis 10S UV–Vis (Thermo Scientific, Waltham, MA, USA).

### 4.5. Biomass Characterization

#### 4.5.1. Protein Content

A Vario EL III elemental analyzer (Vario EL, GmbH, Hanau, Germany) was used to quantify the freeze-dried biomass’s total carbon, hydrogen, and nitrogen (CNH analysis). The biomass (1 mg) was placed in tiny aluminum capsules and heated at 950 °C. Total protein content was calculated by multiplying the nitrogen amount with a conversion factor of 6.25 [45].

#### 4.5.2. Lipid Content

The lipid content of dry biomass was determined using gravimetry after organic extraction followed by the recovery of clear organic phase and further solvent evaporation [46]. The percentage of lipids was calculated with Equation (6):

(6) $$\%\;lipids=100\times \frac{weight\;of\;residue\;from\;evaporated\;clarified\;solvent\;}{weight\;of\;dry\;biomass\;initially\;put\;into\;the\;evaporated\;extractant\;solvent}\;$$

#### 4.5.3. Ash Content

A sample of freeze-dried biomass (50 mg) was weighed in a crucible and taken for combustion at 550 °C for 8 h in a JP Selecta Sel horn R9-L furnace (JP Selecta, 22 Barcelona, Spain). The ash content corresponded to the percentual residual weight of the sample after combustion.

#### 4.5.4. Carbohydrate Content

The carbohydrate content of the dry biomass was calculated as the difference to 100% after summing the percentual contents of the other main components analyzed (protein, ash, and lipid contents).

### 4.6. Statistical Analyses

The statistical tests for OVAT were performed using R software (4.0.2 version) through RStudio 1.3.1073 version (R studio^®^, Boston, MA, USA). ANOVA analysis was followed by a post hoc Tukey HSD test when comparing three or more conditions. A Student’s *t*-test was used to compare groups of independent results. For each test, triplicates, mean, and standard deviation were determined. A statistically significant difference was considered at *p* < 0.05.

The statistical tests for DoE methodology were performed using two software: Minitab (Minitab^®^ version 19, State College, PA, USA), based on a preliminary screening, and Design-Expert (version 12, Stat-Ease^®^, Minneapolis, MN, USA), based on response surface methodology. Minitab was used for a preliminary screening through the Plackett–Burman method followed by Design-Expert Box–Behnken method. Statistical significance was considered at *p* < 0.05 ANOVA tests. The experimentally observed responses were compared with the predicted values (Y) obtained from the model, given by the polynomial Equation (7), correlating the input variables of the study (A, B, and C):

Y = a0 + a1 A + a2 B + a3 C + a4 AB + a5 AC + a6 BC(7)

## Figures and Tables

**Table 1:** Global biomass productivity and specific growth rate of *Scenedesmus rubescens* grown heterotrophically under different pH values. Different letters indicate significant differences, *p* < 0.05. Values are given as average ± standard deviation (*n* = 3).

Conditions (pH)	Global Productivity (g/L/day)	Growth Rate (day^−1^)
6.0	0.94 ± 0.11 ^a^	0.78 ± 0.04 ^a^
6.5	2.95 ± 0.26 ^b^	1.05 ± 0.01 ^b^
7.0	2.98 ± 1.74 ^b^	1.04 ± 0.07 ^b^
8.0	0.30 ± 0.10 ^c^	0.49 ± 0.05 ^c^

**Table 2:** Levels of 4 factors used in DoE (with Design-Expert software, version 12): ammonia, phosphate, nickel, and calcium.

Factors (mM)	Coded Levels		
	Low	Central Point	High
Ammonia (A)	20	40	60
Phosphate (B)	1	5.5	10
Calcium (C)	0.3	1	1.7
Nickel (D)	0	0.01	0.02

**Table 3:** Biomass concentration, global productivity, and specific growth rate of *Scenedesmus rubescens* under different phosphate concentrations. Different letters indicate significant differences between media, *p* < 0.05. Values are given as average ± standard deviation (*n* = 3).

Concentrations (mM)	Biomass Concentration (g/L)	Global Productivity (g/L/ h)	Specific Growth Rate (day^−1^)
10	11.5	0.119 ± 0.012 ^a^	1.18 ± 0.027 ^a^
50	12.2	0.115 ± 0.005 ^ab^	1.17 ± 0.011 ^ab^
100	9.2	0.090 ± 0.011 ^b^	1.09 ± 0.005 ^b^

**Table 4:** Biomass composition at the beginning and end of the cultivation. Proteins, lipids, carbohydrates, and ashes are presented as the percentage of the biomass dry weight. Different letters indicate significant differences between media (*p* < 0.05). Values are given as average ± standard deviation (*n* = 3).

Sample	Proteins (%)	Lipids (%)	Carbohydrates (%)	Ashes (%)
Beginning of cultivation	32.9 ± 0.25 ^a^	13.2 ± 1.90 ^a^	51.4 ± 1.90 ^a^	2.3 ± 0.35 ^a^
End of cultivation	31.2 ± 0.30 ^b^	12.3 ± 1.70 ^a^	53.5 ± 1.40 ^a^	3.2 ± 0.41 ^b^

## Data Availability

Data is available upon request.

## Supplementary Materials

The following supporting information can be downloaded at: https://www.mdpi.com/article/10.3390/md21070411/s1, Table S1: Screening method design in actual level of variables through Minitab^®^ software for *Scenedesmus rubescens*; Table S2: Response functions for optimization of media composition for heterotrophic cultivation of *Scenedesmus rubescens*. Minitab^®^ software, version 19, was used; Table S3: Optimized culture medium developed in this work for 0037SA (macro and micronutrients); Figure S1: Calibration curve. Dry biomass concentration (g L^−1^) vs. absorbance of *S. rubescens* suspensions (in water) measured at λ = 600 nm for heterotrophic growth.
