# Supplementary material for: Scenedesmus rubescens Heterotrophic Production Strategies for Added Value Biomass
Source: Mar Drugs. 2023 Jul 19;21(7):411. doi: 10.3390/md21070411 (PMC10381400; doi:10.3390/md21070411)
Supplement: Supplementary file 1 [file marinedrugs-21-00411-s001.zip › marinedrugs-2499103-supplementary.pdf]

Scenedesmus rubescens heterotrophic production strategies for added value biomass

Gonçalo Espírito Santo [1], Ana Barros[1], Margarida Costa[2], Hugo Pereira[3], Mafalda Trovão[1], Helena Cardoso[1], Bernardo Carvalho[3], Maria Soares[1], Nádia Correia[1], Joana T. Silva[1], Marília Mateus[4] and Joana Silva[1]

[1] Allmicroalgae Natural Products S.A., R&D Department, Rua 25 de Abril s/n, 2445-413 Pataias, Portugal;
[2] Microalgae section, Norwegian Institute for Water Research (NIVA), Økernveien 94, 0579 Oslo, Norway
[3] GreenCoLab—Associação Oceano Verde, University of Algarve, Campus de Gambelas, 8005-139 Faro, Portugal
[4] iBB—Institute for Bioengineering and Biosciences, Instituto Superior Técnico, Universidade de Lisboa, Av. Rovisco Pais, 1049-001 Lisbon, Portugal; marilia.mateus@tecnico.ulisboa.pt

* Correspondence: marilia.mateus@tecnico.ulisboa.pt (M.M.)

Supplementary information

Table S1 - Screening method by Plackett-Burman design in actual level of variables through Minitab® software for *Scenedesmus rubescens*

		Concentration (mM)											
**Trial**	**Nitrogen source**	**N**	**Mg**	**Ca**	**P**	**Fe**	**Cu**	**Zn**	**Mn**	**Mo**	**Co**	**Ni**	**B**
1	Ammonium	40	1.75	1	5.5	0.06	0.03	0.032	0.06	0.011	0.013	0.01	0.3
2	Ammonium	40	1.75	1	5.5	0.06	0.03	0.032	0.06	0.011	0.013	0.01	0.3
3	Nitrate	60	3	0.3	1	0.1	0.06	0.003	0.08	0.020	0.005	0	0.1
4	Nitrate	20	3	0.3	10	0.1	0.06	0.060	0.03	0.001	0.020	0.02	0.1
5	Ammonium	20	0.5	0.3	10	0.02	0.06	0.003	0.08	0.020	0.020	0.02	0.1
6	Nitrate	60	0.5	0.3	10	0.1	0.01	0.060	0.08	0.001	0.005	0	0.1
7	Nitrate	60	0.5	1.7	10	0.02	0.01	0.003	0.03	0.020	0.005	0.02	0.1
8	Ammonium	60	0.5	1.7	10	0.1	0.06	0.003	0.03	0.020	0.020	0	0.5
9	Ammonium	60	3	0.3	10	0.1	0.01	0.003	0.03	0.001	0.020	0	0.5
10	Ammonium	20	3	0.3	10	0.02	0.06	0.060	0.08	0.020	0.005	0	0.5
11	Ammonium	20	0.5	0.3	1	0.02	0.01	0.003	0.03	0.001	0.005	0	0.1
12	Nitrate	40	1.75	1	5.5	0.06	0.03	0.032	0.06	0.011	0.013	0.01	0.3
13	Ammonium	60	3	1.7	10	0.02	0.01	0.060	0.08	0.001	0.020	0.02	0.1
14	Nitrate	20	3	1.7	10	0.1	0.01	0.003	0.08	0.020	0.005	0.02	0.5
15	Nitrate	40	1.75	1	5.5	0.06	0.03	0.032	0.06	0.011	0.013	0.01	0.3
16	Nitrate	20	0.5	1.7	10	0.02	0.06	0.060	0.03	0.001	0.005	0	0.5
17	Nitrate	60	3	1.7	1	0.02	0.06	0.060	0.03	0.020	0.020	0	0.1
18	Nitrate	40	1.75	1	5.5	0.06	0.03	0.032	0.06	0.011	0.013	0.01	0.3
19	Ammonium	40	1.75	1	5.5	0.06	0.03	0.032	0.06	0.011	0.013	0.01	0.3
20	Nitrate	40	1.75	1	5.5	0.06	0.03	0.032	0.06	0.011	0.013	0.01	0.3
21	Ammonium	60	0.5	1.7	1	0.1	0.06	0.060	0.08	0.001	0.005	0.02	0.5
22	Ammonium	20	3	1.7	1	0.1	0.06	0.003	0.03	0.001	0.005	0.02	0.1
23	Ammonium	20	0.5	1.7	1	0.1	0.01	0.060	0.08	0.020	0.020	0	0.1
24	Ammonium	40	1.75	1	5.5	0.06	0.03	0.032	0.06	0.011	0.013	0.01	0.3
25	Ammonium	40	1.75	1	5.5	0.06	0.03	0.032	0.06	0.011	0.013	0.01	0.3
26	Ammonium	40	1.75	1	5.5	0.06	0.03	0.032	0.06	0.011	0.013	0.01	0.3
27	Nitrate	60	0.5	0.3	1	0.02	0.06	0.003	0.08	0.001	0.020	0.02	0.5
28	Ammonium	60	3	0.3	1	0.02	0.01	0.060	0.03	0.020	0.005	0.02	0.5
29	Nitrate	20	3	1.7	1	0.02	0.01	0.003	0.08	0.001	0.020	0	0.5
30	Nitrate	20	0.5	0.3	1	0.1	0.01	0.060	0.03	0.020	0.020	0.02	0.5

Table S2 - Responses functions for screening method composition for heterotrophic cultivation of *Scenedesmus rubescens*. Minitab® software was used

Trials	Biomass concentration (g/L)	Global productivity (g/L/day)	Maximum productivity (g/L/day)
1	9.62	0.089	0.277
2	6.50	0.060	0.142
3	4.41	0.041	0.079
4	12.09	0.112	1.243
5	13.82	0.128	0.344
6	6.90	0.064	0.176
7	10.77	0.100	1.114
8	9.79	0.091	0.166
9	7.31	0.061	0.131
10	7.55	0.070	0.150
11	6.43	0.060	0.142
12	9.62	0.089	0.277
13	11.51	0.091	0.560
14	14.73	0.136	1.235
15	9.21	0.085	0.322
16	12.34	0.114	1.206
17	6.02	0.056	0.109
18	9.21	0.085	0.322
19	6.50	0.060	0.133
20	9.21	0.085	0.322
21	4.33	0.040	0.081
22	5.04	0.047	0.093
23	5.35	0.050	0.120
24	6.91	0.064	0.152
25	6.50	0.060	0.133
26	6.91	0.064	0.152
27	4.91	0.045	0.096
28	3.81	0.035	0.059
29	6.08	0.056	0.212
30	7.53	0.0697	0.150

Table S3 – Optimized culture medium developed in this work for *Scenedesmus rubescens* (macro and micronutrients)

0037 SA medium	
**Component**	Concentration (mM)
**$(NH_4)_2SO_4$**	60
**NaH_2PO_4**	26
**K_2HPO_4**	24
**$MgSO_4.7H_2O$**	1.75
**Citric acid**	6.25
**$FeSO_4.7H_2O$**	0.06
**$CaCl_2.2h_2O$**	0.3
**H_3BO_3**	0.1
**$ZnSO_4$**	0.003
**$MnCl_2.4H_2O$**	0.03
**$Na_2Mo_4.2H_2O$**	0.03
**$CuSO_4.2H_2O$**	0.00325
**$NiCl_2.6H_2O$**	0.02
**$Co(NO_3)_2.6H_2O$**	0.01

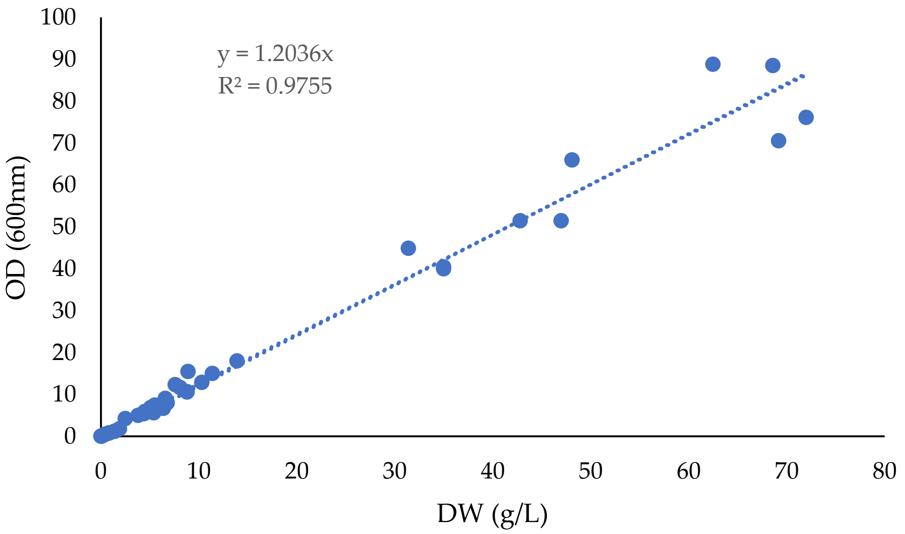

Figure S1 – Calibration Curve. Optical density of *S. rubescens* suspensions (in water) measured at λ= 600 nm versus dry biomass concentration (g/L), for heterotrophic growth.
